# A New Remedy,—Clinkers

**Published:** 1842-04-23

**Authors:** 


### ANALECTA.

*New Remedy.*—Dr. C. J. Edwards, of Bath, in the Provincial Medical and Surgical Journal, Feb. 5th, introduces to the profession a new remedial agent under the name of "*clinkers.*"

"Clinkers" is the refuse of the blacksmith's forge, and differs from common ashes and coke in its greater specific gravity, component parts, and external appearance. As a medicine in cachectic disorders, particularly those of females, it has been used for many years, by "knowing old women" in certain manufacturing districts; and the success which attends its exhibition, particularly in chlorotic disorders, is such as to have won for it the title of "specific."

The following is the formula for its preparation:—The bluest and heaviest clinker, being selected from a mass, is reduced to an impalpable powder (a work of no small difficulty, on account of its metalloid nature). Any quantity of this powder may be mixed, with a sufficiency of treacle to form a stiff paste; and to every eight ounces of the mass half an ounce of magnesia, and the like quantity of ginger must be added. Thus formed, it is any thing but inviting to the eye; but this can be remedied by using honey in lieu of treacle, and adding half a drachm of the peroxide of iron to the compound.

The directions which accompanied the formula were as unique as the formula itself. It must be taken three successive days and nights (twice a day,) for three days; omitted for a like period, and so continued until the course which has been decided upon should be finished. The dose is a teaspoonful. Absurd as these directions seem, they are not so ridiculous as they appear; for experience has demonstrated that constitutional irritation supervenes, unless some decided interval is allowed at stated periods during a course of this remedy.

Dr. Edwards first tested its powers in a number of patients in whom the exsanguineous state of the skin, and wasting of the muscular fibre, were indicative of the morbid manner in which the functions of the stomach, alimentary canal, and uterus were carried on. Within a month from commencing the use of the clinker, a striking improvement took place in the appearance of the patients, and before the termination of two months every unfavorable symptom disappeared. One case in particular is well worthy of especial notice, by reason of the scrofulous condition of the submaxillary glands and the ulcerated state in which one of them had been for several years, and which healed during the course of the new medicine.

I may observe, *en passant*, says Dr. E., that previously to a trial of the clinker, this young woman had been under professional treatment for some time, without deriving any benefit from it.

The pulse was weak, and nearly 100; catamenia irregular in appearance, variable in quantity, and unnatural in composition; appetite and sleep bad; tongue foul; and what perhaps, may be termed hysterical hypochondriasis, existed to a great degree. A gentle cathartic of infusion of senna, with tartrate of potash, was given for a few successive days before the clinker was commenced. She had not taken the new medicine six weeks, when most of the distressing symptoms disappeared, and her personal appearance became so improved, that her friends scarcely recognised in the ruddy girl before them, the pallid and unhealthy creature she had once been.

It appears greatly to benefit cases of simple indigestion, a few doses being capable of removing the most distressing symptoms. In that peculiar condition of the secretion of the bowels, which is said to favour the formation or propagation of worms, it has been proved advantageous in two ways—one, by its mechanical action, the other by its tonic properties. This was an accidental discovery, made during its trial in a case of leucorrhœa.

When the medicine is taken for the first time, a train of symptoms frequently supervenes, which would induce a stranger to its *modus operandi* to regard it as a dangerous compound. A great weight is felt in the epigastric region, accompanied with a burning sensation; sensations of sickness, followed by those of fainting, come on; these are soon relieved by eructations of flatus. Some complain of pains in the limbs, and particularly the joints; others of tightness across the forehead, with giddiness; while all are troubled with heat, dryness of the mouth, and great thirst. At the second dose the symptoms are moderated, and the third is generally taken with impunity. After it has been persevered with for a short time, sensations of a different character arise; these are hunger, and a feeling of health and energy, to which perhaps, the patient has been a stranger for many years; then the complexion, if pale, commonly receives a ruddy tint, and the muscular fibre becomes firm and enlarges. After the first dose the fæces are like pitch, the urine generally pale, and large in quantity; the bowels, if previously costive, become regular in their action; the pulse gets full, and the skin pleasantly relaxed.

To sum up its medicinal properties, it may be said that the clinker is tonic, stimulant, anthelmintic, and colorific, adapted generally to a leucophlegmatic habit, and where dyspeptic, chlorotic, and scrofulous complaints exist; "its use would be contraindicated where an inflammatory diathesis prevails."

The quantity of metal which clinker contains varies considerably; the best is obtained from a blacksmith's forge, and the most ponderous, darkest, and metallic in appearance, is that only on which dependance can be placed. The light slate-colored clinker is inert.

I need not offer any remarks on the magnesia and ginger, which are added in the formation of the linctus; but I may observe that if the ginger be omitted, violent griping ensues. After the medicine has been given for a few weeks, no bad symptoms arise if it is exhibited more frequently than at the beginning of the course.

From the imperfect analysis which I have made of the clinker, I should say that, with the usual substances found in coal partially decomposed by heat, a metalline appearance pervades the mass, which seems to be iron combined with carbon, so as to form steel; no doubt the metal exists likewise, both as sulphuret and a carbonate of the protoxide, but neither of these would afford the blue tint for which the clinker is remarkable. That it is not titanium is evident, for that metal is "like burnished copper," and so little fusible, that the heat of the oxyhydrogen blowpipe, when in action, is scarcely able to touch it. Moreover, after employing all the usual tests, I was unable to detect titanium or its oxide.

That the singularly beneficial effects which the clinker produces in certain conditions of the system cannot result solely from the iron or steel which it contains, the experience we have of those metals and their preparations sufficiently declares; some new combination must exist, to effect such singular changes; what it is remains to be proved. Electricity has been advanced as the cause, but even admitting that galvanic currents could be generated, they would be so weak in power, and small in quantity, as to be incapable of producing any results, while their source would prove so limited, that the electric evolution would cease before the linctus could be formed.

